# Childhood adversities and home atmosphere as determinants of resilience in old age: findings from the Helsinki birth cohort study

**DOI:** 10.1007/s10433-025-00839-z

**Published:** 2025-02-19

**Authors:** Sini Siltanen, Katja Pynnönen, Sini M. Stenroth, Katja Kokko, Markus J. Haapanen, Niko S. Wasenius, Merja K. Laine, Tuija M. Mikkola, Johan G. Eriksson, Mikaela B. von Bonsdorff

**Affiliations:** 1 Folkhälsan Research Center, Helsinki, Finland; 2 Gerontology Research Center and Faculty of Sport and Health Sciences, University of Jyväskylä, Jyvaskyla, Finland; 3 Department of General Practice and Primary Health Care, University of Helsinki and Helsinki University Hospital, Helsinki, Finland; 4 Department of Medical Epidemiology and Biostatistics, Karolinska Institutet, Stockholm, Sweden; 5 Clinicum, Faculty of Medicine, University of Helsinki, Helsinki, Finland; 6 Population Health Unit, Finnish Institute for Health and Welfare, Helsinki, Finland; 7 Department of Obstetrics and Gynecology and Human Potential Translational Research Programme, Yong Loo Lin School of Medicine, National University Singapore, Singapore, Singapore; 8 Institute for Human Development and Potential (IHDP), Agency for Science, Technology and Research (A*STAR), Singapore, Singapore

**Keywords:** Coping, Mediation model, Grip strength, Depressive symptoms, Positive adaptation

## Abstract

Early life stress has far-reaching effects on various aspects of well-being in later life, but whether it impacts resilience, i.e., the ability to tolerate hardship, in old age remains unclear. We investigated whether childhood adversities and childhood home atmosphere are associated with resilience in old age directly or indirectly through poorer physical and psychological functioning in late middle age. The data comprised 1176 persons born in 1934–1944 and were collected over a 17-year follow-up in 2001–2018. Childhood adversities (greater score indicates more adversities) and home atmosphere (greater score indicates better atmosphere) were assessed retrospectively. Resilience in old age was measured with the Hardy-Gill Resilience Scale, depressive symptoms in late middle age with the Beck Depression Inventory, and hand grip strength in late middle age with a dynamometer. Data were analyzed with path modeling with depressive symptoms and grip strength set as mediators. We found that a greater number of childhood adversities and a poorer home atmosphere were associated with poorer resilience in old age (*β* =  − .13; *p* < .001 and *β* = .11, *p* < .001, respectively). These associations were fully mediated by depressive symptoms, but not hand grip strength, in late middle age. The findings indicate that adverse childhood exposures may decrease psychological functioning in middle age, and subsequently, lessen resilience in old age. Future studies should assess whether this pathway can be intervened.

## Introduction

Being exposed to stressful life situations or potentially traumatic events is normal in human life (Bonanno 2004). However, the ways how individuals respond to these events vary considerably. This is partly explained by personal attributes, such as hardiness and self-efficacy, but also by different social and environmental factors (Masten 2001), such as social support, and the type and stressfulness of the adversity (Cosco et al. 2019). Those, who in the face of adversity remain well or even thrive, may be considered as resilient. Resilience refers to adapting to and overcoming adversity and encompasses two critical conditions: first, being exposed to a significant stressor, and second, obtaining a positive outcome (Luthar et al. 2000). It can be identified by examining a person’s response to a specific event, including the event’s initial decremental effect, the time required to regain stability, and the long-term effects of the event (Hardy et al. 2004). In empirical studies, it was found that older people equal or even surpass younger people in resilience (Gooding et al. 2012; MacLeod et al. 2016; Nygren et al. 2005) even though they are more likely to face major losses such as the death of a spouse or a friend and age-related deficits in health and function. However, what leads to resilience in older age, remains underreported.

We believe that childhood experiences have an impact on resilience in older age. In previous studies, it has been found that negative experiences in early life when our bodies and minds are particularly vulnerable and still developing, can have far-reaching consequences in various aspects of well-being (Parvin et al. 2024). It has been suggested that early life stress may disturb the normal development of the prefrontal cortex, hippocampus, hypothalamus, and amygdala and produce unwanted hormonal and neuropeptide alterations (Smith & Pollak 2020). In later life, altered activity of these systems may be manifested as weakened behavioral responses to threats and challenges and be associated with negative mental health and physical health outcomes (Smith & Pollak 2020), i.e. lower resilience. Furthermore, early life stress can negatively affect emotional regulation, trust, and communication, and subsequently, increase the tendency for social isolation (Parvin et al. 2024), negative social interactions, and conflict in romantic relationships (Luecken et al. 2013). These developmental challenges may also underlie poorer resilience as social support, social interaction, self-efficacy, and hardiness are its important attributes (MacLeod et al. 2016). Moreover, negative childhood experiences, such as neglect, abuse, poor socioeconomic situation, or war separation, have been associated with higher rates of depression, anxiety, obesity, reduced physical activity, accelerated aging, frailty, and various chronic conditions, such as heart disease and diabetes in old age (Haapanen et al. 2022a; Parvin et al. 2024; Smith & Pollak 2020). Furthermore, most people who have experienced one adversity in their childhood, e.g., parents’ divorce, may be disproportionately affected by other risks too, for instance, economic hardship, moving, and chronic family conflict (Masten et al. 2011). Such cumulation of negative life experiences in childhood, or stress proliferation as referred to in some occasions (Pearlin et al. 2005), may lessen the ability to tolerate and overcome hardship in later life, i.e., negatively contribute to resilience.

The pathway from negative childhood exposures to poorer resilience in later life may be mediated by poorer physical and psychological functioning. We previously reported that childhood adversities, such as poverty, parental divorce and substance abuse, and childhood home atmosphere, describing how supportive, caring, and warm it was, are associated with physical and psychological functioning in old age, such that adversities coincide with poorer functioning, whereas a better home atmosphere relates to better functioning (von Bonsdorff et al. 2019). Similar findings regarding childhood adversities have also been reported elsewhere (e.g. Alastalo et al. 2013; Campbell et al. 2016; Mäkinen et al. 2006). Good physical and psychological functioning, in turn, are established key elements of successful aging (Rowe & Kahn 1997), and hence may contribute to resilience in old age. For example, better physical performance (Koivunen et al. 2020, 2022; Tourunen et al. 2020) and fewer depressive symptoms (Hardy et al. 2004; Tourunen et al. 2021) have been associated with better coping ability and greater resilience in older age, and vice versa.

To understand resilience in aging more thoroughly, we aimed to study first, whether childhood adversities and childhood home atmosphere are associated with resilience in old age, and second, whether psychological and physical functioning in late middle age mediate these associations and thus, function as a plausible mechanism explaining the associations between childhood adversities and resilience in old age. Depressive symptoms were used as a marker of psychological function and hand grip strength as a marker of physical function, as they are well-established variables and easy to assess in large population-based studies. In addition, hand grip strength and depression are highly predictive of functional deficits and disability (Rantakokko et al. 2013; Rantanen et al. 1999; WHO 2023; Vaishya et al. 2024).

## Methods

### Data and participants

We used data from the Helsinki Birth Cohort Study (HBCS), which comprises 8760 women and men born in the Helsinki University Central Hospital, Finland, between the years 1934 and 1944. The HBCS data have been collected in multiple subsequent clinical examinations and by postal surveys between the years 2001 and 2018 and are complemented with information from several national registers regarding, e.g., hospital admissions, socio-economic factors, and deaths. Flow chart of the HBCS study has been introduced earlier (Haapanen et al. 2022b). Briefly, a random sample (*N* = 2904) of the birth cohort was invited to participate in the first comprehensive clinical examinations at baseline in 2001–2004. Of them, 2003 individuals (mean age = 61.5 years, standard deviation (SD) = 2.9 years) consented to participate. Follow-up clinical visits were conducted for those individuals who were still alive and living within a 100 km distance from the study clinic in Helsinki in 2011–2013 and 2017–2018 (invited *N* = 1404, participated *N* = 1094 and invited *N* = 1174, participated *N* = 815, respectively). In addition to the clinical cohort, a questionnaire was mailed to all surviving cohort members (*n* = 1577) in 2015. Of them, 1153 individuals responded. The present study utilizes information from the clinical visits conducted at baseline in 2001–2004 and at follow-up in 2017–2018, and from the postal survey in 2015. A total of 1176 study participants had data on childhood exposures, resilience, depressive symptoms, and hand grip strength at baseline, and thus, formed the sample for the current study.

### Measures

*Resilience* in old age was assessed in 2015 or 2017–18 (mean age 75.9 years in the latter) with the validated Hardy-Gill resilience scale (Hardy et al. 2002, 2004), which consists of three independent components: (1) identifying the most stressful adverse event in the past 5 years, (2) rating the level of stressfulness of the identified event and (3) assessing one’s subjective experiences of the event, their recovery, and the consequences of the event with structured questions. The cohort members were first asked to identify the most stressful life event during the past five years in an open-ended question. Subsequently, they were asked to rate its level of stressfulness on a visual analog scale, making a mark along a continuous 140 mm line from “not particularly” to “extremely”. To determine the exact value, the length of the line was measured from the start to the point the respondent marked, with a higher value indicating greater stressfulness of the adverse event. Finally, nine structured questions were asked based on the resilience module of the Asset and Health Dynamics (AHEAD) Study (Soldo et al. 1997). These items were, e.g., “After this event, how much more discouraged were you?”, “As a result of this event, have you stopped doing some activities that were important to you?”, and “Has this event made a permanent change in how you feel about your life?”. The response options (e.g. ranging from a great deal to not at all) were transformed to form a total score ranging from 0 (least resilient) to 18 (most resilient). A more detailed description of the procedure and scale can be found elsewhere (Hardy et al. 2004). A member of the research group translated the scale from English into Finnish, after which the translation was carefully discussed in a research group meeting. The researchers involved in this process were familiar with the concept of resilience, native Finnish speakers, and fluent in English, with some being employed in academia in English-speaking countries. Finally, a bilingual third-party person with both English and Finnish as a mother tongue back-translated the scale, and the translation was again carefully examined until a consensus was achieved. In the present study, we used the third component (a scale ranging from 0 to 18) as a marker of resilience. In case the participant had data on resilience both in 2015 and 2017–2018, we considered only the period that the participant had rated as more stressful in the second component. For examining the descriptive characteristics of participants, resilience was divided into data-driven tertiles: lowest (0–9), intermediate (10–13), and highest (14–18). Otherwise, the variable was used as continuous.

*Childhood adversities and home atmosphere* were assessed retrospectively at baseline in 2001–2004. First, the participants reported whether they had experienced the following adversities under the age of 16: long-term financial difficulties, unemployment of one parent, serious illness of a parent, father’s alcohol problem, mother’s alcohol problem, father’s psychiatric illness (e.g., schizophrenia, depression), mother’s psychiatric illness, serious conflict in the family, parental divorce, own serious or long-term illness, or having been bullied at school (Korkeila et al. 2010). In addition, information on separations during World War II, in which children (cohort members) were sent abroad unaccompanied by their parents, was obtained from the Finnish National Archives (Pesonen et al. 2010). One point was given to each adversity the participant reported on, resulting in a sum score ranging from 0 (minimum) to 12 (maximum). Secondly, the participants were asked to rate nine features of their home atmosphere*.* These features were warm, caring, inspiring, supportive, quarrelsome, trusting, understanding, strict, open, unfair, happy, and uninterested. Response options ranged on a 5-point scale from 0 (does not describe) to 4 (describes exactly). Negative features were reverse scored and subsequently, all scores were summed (range 0–36), with higher scores indicating a better home atmosphere (von Bonsdorff et al. 2019). Cronbach’s alpha for home atmosphere was 0.88.

*Mediating variables* were depressive symptoms and hand grip strength, and they were assessed in late middle age at the clinical baseline in 2001–2004 (mean age 61.3 years). Depressive symptoms were measured with the modified version of the Beck Depression Inventory (Beck et al. 1974), BDI-IA. Each of the 21 items was rated from 0 to 3 and summed (range 0–63) with a higher score indicating greater severity of symptoms. Isometric grip strength of the dominating hand was measured with a Newtest Grip Force dynamometer (Newtest Oy, Oulu, Finland) (Ylihärsilä et al. 2007). The maximum value of three squeezes was used in the present analyses.

*Covariates* included age at baseline, sex, marital status at follow-up (assessed at the same time as resilience), and stressfulness of an adverse event (assessed at the same time as resilience), as these variables had a statistically significant association with at least one of the main variables and they improved the mediation model fit. In addition, the stressfulness of an adverse event was seen to provide additional information about resilience, as the type, meaning, and magnitude of adversity typically impact the way an individual responds to it (Cosco et al. 2019). Marital status was dichotomous “married” versus “not married”. Stressfulness of an adverse event was assessed as an independent part of the Hardy-Gill resilience scale. Furthermore, we considered socioeconomic status (SES) in childhood and educational attainment in late middle age as plausible covariates, but neither of them was associated with the predictors or the outcome and including either of them in the analysis deteriorated the model fit. Hence, these two variables were excluded from the final model.

### Statistical analysis

**Fig. 1:**
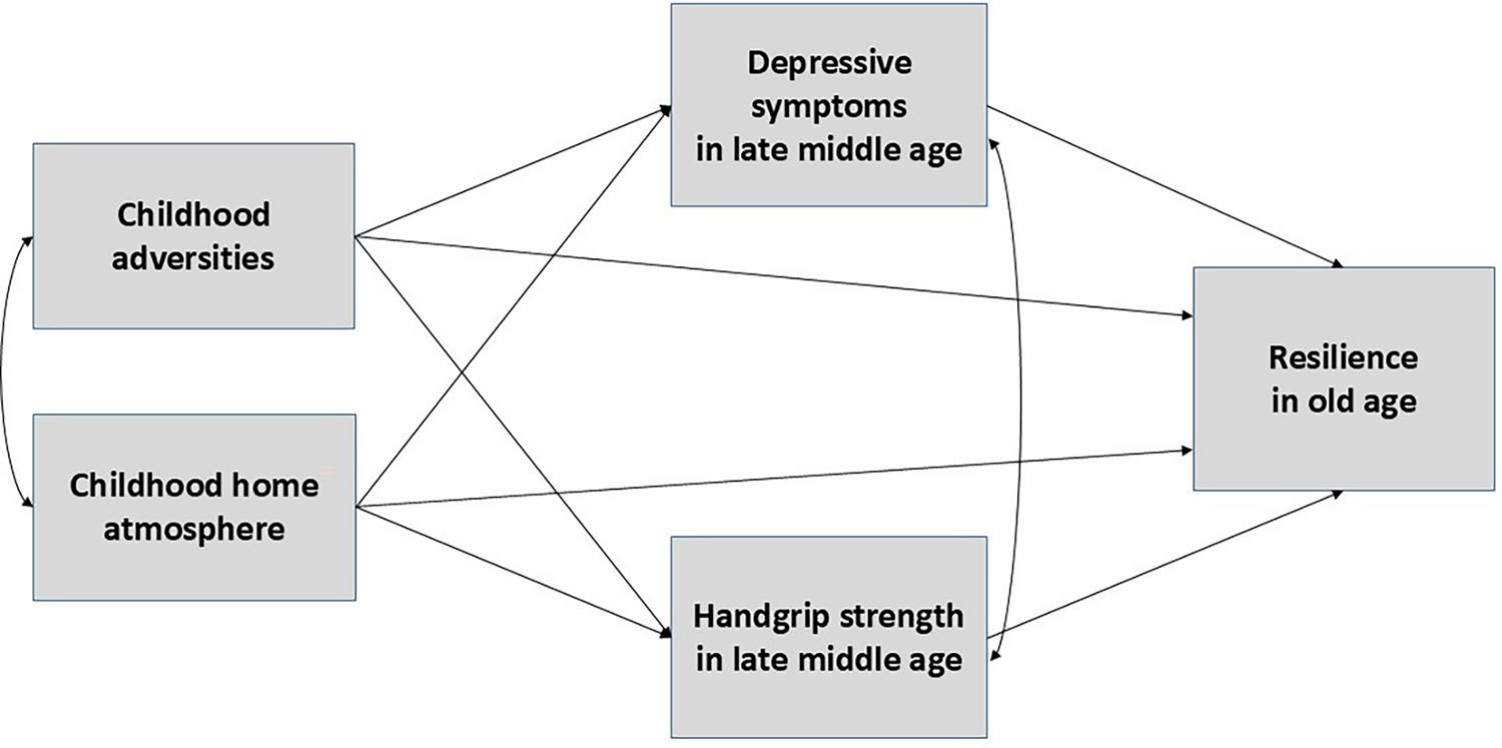
The conceptual model for the mediation analysis with depressive symptoms and handgrip strength in late middle age set as mediators of the associations between childhood adversities, childhood home atmosphere (predictors), and resilience in old age (outcome). *Note*: Additionally, the model included sex, age at baseline, marital status at follow-up, and stressfulness of an adverse event at follow-up as covariates

The descriptive data of study participants by resilience tertiles were investigated using means, standard deviations and percents, and the differences between these analyzed with one-way ANOVA, Bonferroni post hoc test (continuous variables) and chi-square test (categorical variables). Then, to study the total effect, i.e., the association between childhood adversities, childhood home atmosphere, and resilience in old age, we used bivariate regression analyses. Childhood adversities and home atmosphere were analyzed in separate models. Finally, the mediation effects were analyzed with path modeling according to Fig. 1, with all main variables and covariates included in the model simultaneously.

The analyses were conducted with MPLUS (version 8.6) (Muthén & Muthén, 2012) using maximum likelihood robust (MLR) as an estimator to obtain parameter estimates. MLR produces robust scale-corrected chi-square values and robust standard errors, which correct bias in case of non-normally distributed data and heteroscedasticity. In the present study, the distribution of depressive symptoms was right skewed, and hence it was square root-transformed before the analysis. One to three missing items were allowed in each sum scale (resilience, depressive symptoms, childhood adversities, and home atmosphere) and imputed using the multiple imputation procedure of SPSS Statistics (version 26) based on the available information on the respective scale. After the multiple imputation procedure executed with SPSS, the amount of missing data varied between 0.1% and 6.5% in all study variables. The remaining missing data were imputed in the path modeling phase by using the MPLUS estimator adapted for missing at random (MAR). There were no missing data on age and sex. The chi-square test value, root mean square error of approximation (RMSEA < 0.06), comparative fit index (CFI > 0.95), Tucker-Lewis index (TLI > 0.95), and standardized root mean residual (SRMR < 0.08) were used as fit indices to explore the suitability of the model to the observed data (Hu & Bentler 1999).

## Results

Descriptive characteristics of the study participants are shown in Table 1. The mean age at baseline was 61.3 years (standard deviation, SD = 2.8 years), and of the cohort, 56% were women and 67% were married at the time when resilience was assessed. Participants belonging to the highest resilience tertile were younger, and a greater proportion of them were married and men compared to people in the lowest resilience tertile. In addition, people in the highest resilience tertile demonstrated the most positive outcomes in the other background variables, e.g., they had fewer childhood adversities, better home atmosphere, fewer depressive symptoms, and better hand grip strength than those in the lowest resilience tertile. In addition, those in the highest resilience tertile had better hand grip strength and less stressful adversities compared to those in the intermediate resilience tertile.

**Table 1:** Descriptive data of the study participants by resilience tertiles

		Tertiles of resilience			
	All (*N* = 1176)	Highest (*N* = 340)	Intermediate (*N* = 429)	Lowest (*N* = 404)	
	Mean (SD)	Mean (SD)	Mean (SD)	Mean (SD)	*p *value
Age (years)^1^	61.3 (2.8)	60.9 (2.5)	61.1 (2.7)	61.7 (3.0)	< .001^3^
Women (%, N)^1^	56 (660)	43 (147)	54 (232)	69 (279)	< .001^4^
Married (%, N)^2^	67 (792)	77 (260)	69 (293)	59 (237)	< .001^4^
*Childhood*					
Number of childhood adversities (0–12)^1^	1.8 (1.7)	1.6 (1.7)	1.8 (1.7)	2.0 (1.7)	< .001^3^
Home atmosphere in childhood (0–36)^1^	24.3 (6.7)	25.0 (6.7)	24.6 (6.3)	23.4 (6.9)	.002^3^
*Late middle age*					
Depressive symptoms in late middle age (0–63)^1^	5.4 (4.9)	4.1 (4.5)	4.8 (4.4)	7.0 (5.3)	< .001^3^
Handgrip strength in late middle age (kg)^1^	31.6 (12.0)	34.4 (12.4)	32.0 (11.5)	28.7 (11.5)	< .001^3^
*Old age*					
Resilience in old age (0–18)^2^	10.7 (3.9)	–	–	–	–
Stressfulness of adversity in old age (0–140)^2^	102.2 (31.7)	81.2 (34.8)	102.5 (27.0)	119.2 (21.6)	< .001^3^

^1^assessed at baseline in 2001–2004, ^2^assessed at follow-up in 2015 or 2017–2018, ^3^tested with ANOVA, ^4^tested with chi square test. Cutoff points for the resilience tertiles were lowest 0–9, intermediate 10–13, highest 14–18. The resilience score was missing for three participants and imputed not until in the path modeling phase

Correlations between the main study variables were rather small but statistically significant (Table 2). Both childhood adversities and home atmosphere had an association with resilience in old age. A greater number of childhood adversities was associated with poorer resilience in old age whereas a better home atmosphere in childhood was associated with greater resilience in old age (standardized path coefficient, *β* =  − 0.13; 95% CI − 0.19, − 0.07; *p* < 0.001 and *β* = 0.11; 95% CI 0.05, 0.18; *p* < 0.001, respectively).

**Table 2:** Correlation matrix of the study variables tested with Spearman’s correlation

	1	2	3	4	5	6	7	8	9
1 Resilience in old age	1								
2 Childhood adversities	− 0.13**	1							
3 Childhood home atmosphere	0.11**	− 0.45**	1						
4 Depressive symptoms in late middle age	− 0.28**	0.19**	− 0.23 **	1					
5 Handgrip strength in late middle age	0.21**	− 0.10**	0.09**	− 0.22**	1				
6 Stressfulness of adversity in old age	− 0.53**	0.13**	− 0.04	0.18**	− 0.23**	1			
7 Age at baseline	− 0.13**	0.07	− 0.01	0.05	− 0.16**	− 0.01	1		
8 Sex (men vs. women)	− 0.21**	0.09**	− 0.05	0.20**	− 0.79**	0.26**	− 0.08	1	
9 Married at follow-up (no vs. yes)	0.15**	− 0.04	0.01	− 0.10**	0.28**	− 0.15**	− 0.08*	− 0.31**	1

* =  < .05; ** =  < .01

**Fig. 2:**
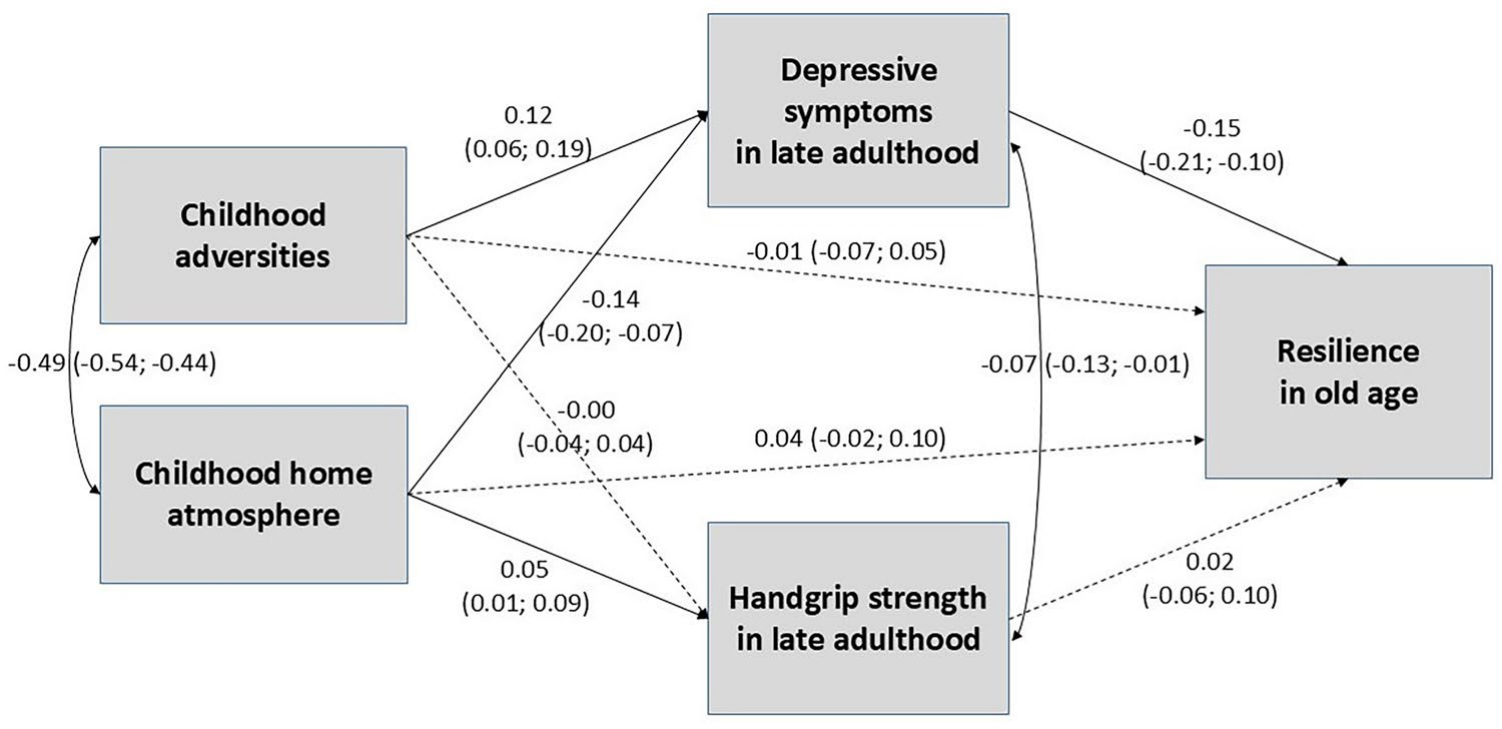
Standardized parameter estimates and 95% confidence intervals for the associations in the adjusted mediation path model. *Note*: Statistically significant associations are marked in bold arrows and non-significant associations are in dashed arrows. The model was adjusted for sex, age at baseline, marital status at follow-up, and stressfulness of an adverse event at follow-up. *R*.^*2*^ = 0.29. Fit indices for the model: (χ2(12) = 18.298, p = .0816; RMSEA = .023; CFI = .995; TLI = .990; and SRMR = .023)

The path model showed that when depressive symptoms and hand grip strength in late middle age were added to the model as mediators, the direct associations between childhood exposures and resilience in old age became statistically non-significant (Fig. 2). This indicated that a full mediation effect between the studied variables could be detected. A higher number of childhood adversities was associated with more depressive symptoms in late middle age, whereas a better childhood home atmosphere was associated with less depressive symptoms in late middle age. Depressive symptoms, in turn, were associated with poorer resilience in old age. Furthermore, a better home atmosphere in childhood was associated with greater hand grip strength in late middle age, but hand grip strength was not associated with resilience in old age. Thus, no mediation effect was found for hand grip strength. Altogether, the path model explained 29% of the variance in resilience in old age. The model also fitted the data well (χ2(9) = 16.964, *p* = 0.049; RMSEA = 0.027; CFI = 0.994; TLI = 0.987; and SRMR = 0.023).

## Discussion

The findings of this study suggest that childhood adversities and home atmosphere are associated with resilience in old age, such that a greater number of childhood adversities and a poorer home atmosphere relate to lower resilience in old age. This association is explained by a higher prevalence of depressive symptoms, but not poorer hand grip strength in late middle age. In line with previous research (Alastalo et al. 2013; Campbell et al. 2016; Mäkinen et al. 2006; von Bonsdorff et al. 2019), the present findings emphasize the influence of childhood exposures on psychological function in later life but expand the previous knowledge to cover the process of psychological adaptation and the ability to tolerate hardship, i.e., resilience.

Based on the findings of this study, negative childhood exposures, here including childhood adversities and poor home atmosphere, are associated with poorer ability to tolerate hardship in old age. This is partly in contradiction with previous findings noting that a history of some adversity may promote the ability to tolerate further hardship, also called the “steeling” effect that decreases one’s vulnerabilities (Rutter 2012). On the other hand, it has also been suggested that adversities tend to cumulate from childhood onward (Masten et al. 2011; Pearlin et al. 2005). In the present study, 48% (*n* = 567) of the participants reported on more than one adversity in their childhood whereas 27% (*n* = 315) reported on no adversities and 25% (*n* = 293) on one adversity. Cumulated adversities, unlike single stressors, have been linked to an increased risk of psychopathology (Evans et al. 2013), higher global distress, functional impairment, posttraumatic stress symptoms, and lower life satisfaction (Seery et al. 2010). The accumulation of adversities may hinder the development of certain resources and capacity needed to overcome and adapt to adversity, which may explain the present findings. For example, it has been suggested that early life stress might contribute to psychopathology in later life by impairing some physiological stress response systems, e.g. cortisol reactivity, emotion regulation, and cognitive function, such that they may function sub-optimally under stress (Cărnuţă et al. 2015; Hedges & Woon 2011; Pechtel & Pizzagalli 2011; Pesonen & Räikkönen 2012). Furthermore, it has been shown that although some degree of recovery in functioning may be achieved after the early life stress has ceased, deficits in emotion regulation and affective functioning, in particular, seem to persist into later life (Pechtel & Pizzagalli 2011).

We observed a full mediation effect of depressive symptoms, but not hand grip strength, in late middle age on the associations between childhood adversities, home atmosphere, and resilience in old age. Thus, depression may be considered as a plausible mechanism explaining the relationship between negative childhood exposures and lower resilience in old age. The link between negative childhood exposures and depressive symptoms in adulthood has been recognized also previously (Campbell et al. 2016; Korkeila et al. 2010; Pesonen & Räikkönen 2012). Depressive symptoms, in turn, have been linked to lower resilience in older age, and vice versa (Hardy et al. 2004; Tourunen et al. 2021). These findings may partially be explained by the stress generation theory. It posits that depressive symptoms and stress generate a cycle, where people who experience stress, later experience depressive symptoms and yet again an excess of stressors, partly because of their personal characteristics and behavior, but also due to history with stressors (Hammen 2006). Especially neglect, lack of supportive relationships and abuse in childhood have been linked to maladaptive behavior, and subsequently, depression in later life (Harris 2001). In the present study, the most common adversities reported in childhood were economic deprivation, father’s alcoholism, family conflicts, and parent’s illness or disability, which all may imply an ignorant or unsupportive environment. Hence, for these persons in particular, achieving the key attributes of resilience, such as greater self-efficacy, positive self-concept (Gillespie et al. 2007; Hicks & Conner 2014), and the ability to solve problems, look to the future, and use humor (Resnick 2014; van Kessel 2013) may be difficult.

Physical functioning, here operationalized as hand grip strength, was not found to mediate the association between childhood exposures and resilience in old age although there is evidence that childhood exposures influence physical functioning in later life (Alastalo et al. 2013; von Bonsdorff et al. 2019) and that physical functioning relates to resilience in old age (Koivunen et al. 2020, 2022; Tourunen et al. 2020). Hence, our findings indicate that instead of being a plausible mechanism underlying the associations, hand grip strength may have solely independent associations with childhood exposures and resilience. However, this assumption merits further inspection.

To our best knowledge, no empirical study has thus far viewed older people’s resilience from a life-course perspective incorporating direct and indirect associations between childhood exposures, functioning in late middle age, and resilience in old age. Hence, the present study adds value to earlier literature by addressing not only the temporal order of associations but also the underlying mechanisms and thus, generates ground for new causal hypotheses regarding resilience in aging. In addition, we used validated measures including both self-report and objective assessments, and the data were reasonable in size and comprised both men and women. However, some limitations should also be mentioned. First, the childhood exposures were assessed retrospectively and based on self-reports, which might entail recall bias. In another study, however, it was found that siblings similarly recalled parenting styles from their childhood, which may support the reliability of these types of data (McCrae & Costa Jr, 1988). Second, in the present data, the average number of adversities in childhood was relatively low and home atmosphere, in turn, relatively good. Hence, as the participants of this study came from rather good childhood homes and were not exposed to a lot of hardship, we cannot rule out the possibility that the effects of negative childhood exposures are underestimated here.

## Conclusions

In conclusion, we found evidence of the longitudinal associations between childhood adversities, poorer home atmosphere, and lower resilience in old age, with depressive symptoms in late middle age being a plausible mechanism underlying these associations. The present findings highlight the influence of negative childhood exposures on psychological function in later life and imply that successful aging and adaptation to age-related losses may be hindered if a person is exposed to early life stress and depression in late middle age. Moreover, hand grip strength was not found to mediate the associations between childhood exposures and resilience in old age, indicating that physical function may have solely independent associations with these two. Future studies should investigate whether the path from negative childhood exposures to poorer resilience through poorer psychological well-being could be intervened in order to promote successful aging.

## Data Availability

Pseudonymized data are available to external collaborators upon agreement on the terms of data use and publication of results. To request the data, please contact Senior Researcher Tuija Mikkola (tuija.mikkola@folkhalsan.fi).

## References

[CR1] Alastalo H, von Bonsdorff MB, Räikkönen K, Pesonen A-K, Osmond C, Barker DJP, Heinonen K, Kajantie E, Eriksson JG (2013) Early life stress and physical and psychosocial functioning in late adulthood. PLoS ONE 8(7):e69011. 10.1371/journal.pone.0069011 23861956 10.1371/journal.pone.0069011 PMC3702583

[CR2] Beck AT, Rial WY, Rickels K (1974) Short form of depression inventory: cross-validation. Psychol Rep 34(3_suppl):1184–1186. 10.1177/003329417403403s01 4424377

[CR3] Bonanno GA (2004) Loss, trauma, and human resilience: have we underestimated the human capacity to thrive after extremely aversive events? Am Psychol 59(1):20–28. 10.1037/0003-066X.59.1.20 14736317 10.1037/0003-066X.59.1.20

[CR4] Campbell JA, Walker RJ, Egede LE (2016) Associations between adverse childhood experiences, high-risk behaviors, and morbidity in adulthood. Am J Prev Med 50(3):344–352. 10.1016/j.amepre.2015.07.022 26474668 10.1016/j.amepre.2015.07.022 PMC4762720

[CR5] Cărnuţă M, Crişan LG, Vulturar R, Opre A, Miu AC (2015) Emotional non-acceptance links early life stress and blunted cortisol reactivity to social threat. Psychoneuroendocrinology 51:176–187. 10.1016/j.psyneuen.2014.09.026 25462891 10.1016/j.psyneuen.2014.09.026

[CR6] Cosco TD, Kok A, Wister A, Howse K (2019) Conceptualising and operationalising resilience in older adults. Health Psychol Behav Med 7(1):90–104. 10.1080/21642850.2019.1593845 34040841 10.1080/21642850.2019.1593845 PMC8114384

[CR7] Evans GW, Li D, Whipple SS (2013) Cumulative risk and child development. Psychol Bull 139(6):1342–1396. 10.1037/a0031808 23566018 10.1037/a0031808

[CR8] Gillespie BM, Chaboyer W, Wallis M (2007) Development of a theoretically derived model of resilience through concept analysis. Contemp Nurse 25(1–2):124–135. 10.5172/conu.2007.25.1-2.124 17622996 10.5172/conu.2007.25.1-2.124

[CR9] Gooding PA, Hurst A, Johnson J, Tarrier N (2012) Psychological resilience in young and older adults. Int J Geriatr Psychiatry 27(3):262–270. 10.1002/gps.2712 21472780 10.1002/gps.2712

[CR10] Haapanen MJ, Jylhävä J, Kortelainen L, Mikkola TM, Salonen M, Wasenius NS, Kajantie E, Eriksson JG, von Bonsdorff MB (2022) Early-life factors as predictors of age-associated deficit accumulation across 17 years from midlife into old age. J Gerontol: Series A 77(11):2281–2287. 10.1093/gerona/glac007 10.1093/gerona/glac007 PMC9678199 35018457

[CR11] Hammen C (2006) Stress generation in depression: reflections on origins, research, and future directions. J Clin Psychol 62(9):1065–1082. 10.1002/jclp.20293 16810666 10.1002/jclp.20293

[CR12] Hardy SE, Concato J, Gill TM (2002) Stressful life events among community-living older persons. J Gen Intern Med 17(11):841–847. 10.1046/j.1525-1497.2002.20105.x 10.1046/j.1525-1497.2002.20105.x PMC1495128 12406354

[CR13] Hardy SE, Concato J, Gill TM (2004) Resilience of community-dwelling older persons. J Am Geriatr Soc 52(2):257–262. 10.1111/j.1532-5415.2004.52065.x 14728637 10.1111/j.1532-5415.2004.52065.x

[CR14] Harris T (2001) Recent developments in understanding the psychosocial aspects of depression. Br Med Bull 57(1):17–32. 10.1093/bmb/57.1.17 11719916 10.1093/bmb/57.1.17

[CR15] Hedges DW, Woon FL (2011) Early-life stress and cognitive outcome. Psychopharmacology 214(1):121–130. 10.1007/s00213-010-2090-6 21107538 10.1007/s00213-010-2090-6

[CR16] Hicks MM, Conner NE (2014) Resilient ageing: a concept analysis. J Adv Nurs 70(4):744–755. 10.1111/jan.12226 23919385 10.1111/jan.12226

[CR17] Hu L, Bentler PM (1999) Cutoff criteria for fit indexes in covariance structure analysis: conventional criteria versus new alternatives. Struct Equ Model 6(1):1–55. 10.1080/10705519909540118

[CR18] Koivunen K, Sillanpää E, von Bonsdorff M, Sakari R, Törmäkangas T, Rantanen T (2020) Mortality risk among older people who did versus did not sustain a fracture: baseline prefracture strength and gait speed as predictors in a 15-year follow-up. J Gerontol: Series A 75(10):1996–2002. 10.1093/gerona/glz251 10.1093/gerona/glz251 31628484

[CR19] Koivunen K, Portegijs E, Sillanpää E, Eronen J, Kokko K, Rantanen T (2022) Maintenance of high quality of life as an indicator of resilience during COVID-19 social distancing among community-dwelling older adults in Finland. Qual Life Res 31(3):713–722. 10.1007/s11136-021-03002-0 34570331 10.1007/s11136-021-03002-0 PMC8475423

[CR20] Korkeila J, Vahtera J, Nabi H, Kivimäki M, Korkeila K, Sumanen M, Koskenvuo K, Koskenvuo M (2010) Childhood adversities, adulthood life events and depression. J Affect Disord 127(1):130–138. 10.1016/j.jad.2010.04.031 20569993 10.1016/j.jad.2010.04.031

[CR21] Luecken LJ, Roubinov DS, Tanaka R (2013) Childhood family environment, social competence, and health across the lifespan. J Soc Pers Relat 30(2):171–178. 10.1177/0265407512454272

[CR22] Luthar SS, Cicchetti D, Becker B (2000) The construct of resilience: a critical evaluation and guidelines for future work. Child Dev 71(3):543–562 10953923 10.1111/1467-8624.00164 PMC1885202

[CR23] MacLeod S, Musich S, Hawkins K, Alsgaard K, Wicker ER (2016) The impact of resilience among older adults. Geriatr Nurs 37(4):266–272. 10.1016/j.gerinurse.2016.02.014 27055911 10.1016/j.gerinurse.2016.02.014

[CR24] Mäkinen T, Laaksonen M, Lahelma E, Rahkonen O (2006) Associations of childhood circumstances with physical and mental functioning in adulthood. Soc Sci Med 62(8):1831–1839. 10.1016/j.socscimed.2005.08.040 16194591 10.1016/j.socscimed.2005.08.040

[CR25] Masten AS (2001) Ordinary magic: resilience processes in development. Am Psychol 56(3):227–238. 10.1037/0003-066X.56.3.227 11315249 10.1037//0003-066x.56.3.227

[CR26] Masten AS, Monn AR, Supkoff LM (2011) Resilience in children and adolescents. In: Southwick SM, Litz BT, Charney D, Friedman MJ (eds) Resilience and mental health: challenges across the lifespan. Cambridge University Press, pp 103–119. 10.1017/CBO9780511994791.009

[CR27] McCrae RR, Costa PT Jr (1988) Recalled parent-child relations and adult personality. J Pers 56(2):417–434. 10.1111/j.1467-6494.1988.tb00894.x 3404384 10.1111/j.1467-6494.1988.tb00894.x

[CR28] Muthén, LK, & Muthén, BO (2012) *Mplus user’s guide* (6th Edition)

[CR29] Nygren B, Aléx L, Jonsén E, Gustafson Y, Norberg A, Lundman B (2005) Resilience, sense of coherence, purpose in life and self-transcendence in relation to perceived physical and mental health among the oldest old. Aging Ment Health 9(4):354–362. 10.1080/1360500114415 16019292 10.1080/1360500114415

[CR30] Parvin MR, Johra FT, Akter F, Wahiduzzaman MD, Akter K, Das M, Mondal S, Debnath M, Ullah M, Rony MKK (2024) The long-term effects of childhood circumstances on older individuals: s systematic review. Aging Med 7(2):239–251. 10.1002/agm2.12299 10.1002/agm2.12299 PMC11077334 38725695

[CR31] Pearlin L, Schieman S, Fazio E, Meersman S (2005) Stress, health, and the life course: some conceptual perspectives. J Health Soc Behav 46:205–219. 10.1177/002214650504600206 16028458 10.1177/002214650504600206

[CR32] Pechtel P, Pizzagalli DA (2011) Effects of early life stress on cognitive and affective function: an integrated review of human literature. Psychopharmacology 214(1):55–70. 10.1007/s00213-010-2009-2 20865251 10.1007/s00213-010-2009-2 PMC3050094

[CR33] Pesonen A-K, Räikkönen K (2012) The lifespan consequences of early life stress. Physiol Behav 106(5):722–727. 10.1016/j.physbeh.2011.10.030 22079583 10.1016/j.physbeh.2011.10.030

[CR34] Pesonen A-K, Räikkönen K, Feldt K, Heinonen K, Osmond C, Phillips DIW, Barker DJP, Eriksson JG, Kajantie E (2010) Childhood separation experience predicts HPA axis hormonal responses in late adulthood: a natural experiment of World War II. Psychoneuroendocrinology 35(5):758–767. 10.1016/j.psyneuen.2009.10.017 19963324 10.1016/j.psyneuen.2009.10.017

[CR35] Rantakokko M, Mänty M, Rantanen T (2013) Mobility decline in old age. Exerc Sport Sci Rev 41(1):19. 10.1097/JES.0b013e3182556f1e 23038241 10.1097/JES.0b013e3182556f1e

[CR36] Rantanen T, Guralnik JM, Foley D, Masaki K, Leveille S, Curb JD, White L (1999) Midlife hand grip strength as a predictor of old age disability. JAMA 281(6):558–560. 10.1001/jama.281.6.558 10022113 10.1001/jama.281.6.558

[CR37] Resnick B (2014) Resilience in older adults. Topics Geriatric Rehabilitation 30(3):155. 10.1097/TGR.0000000000000024

[CR38] Rowe JW, Kahn RL (1997) Successful aging1. Gerontologist 37(4):433–440. 10.1093/geront/37.4.433 9279031 10.1093/geront/37.4.433

[CR39] Rutter M (2012) Resilience as a dynamic concept. Dev Psychopathol 24(2):335–344. 10.1017/S0954579412000028 22559117 10.1017/S0954579412000028

[CR40] Seery MD, Holman EA, Silver RC (2010) Whatever does not kill us: cumulative lifetime adversity, vulnerability, and resilience. J Pers Soc Psychol 99:1025–1041. 10.1037/a0021344 20939649 10.1037/a0021344

[CR41] Smith KE, Pollak SD (2020) Early life stress and development: potential mechanisms for adverse outcomes. J Neurodev Disord 12(1):34. 10.1186/s11689-020-09337-y 33327939 10.1186/s11689-020-09337-y PMC7745388

[CR42] Soldo BJ, Hurd MD, Rodgers WL, Wallace RB (1997) Asset and health dynamics among the oldest old: an overview of the AHEAD study. J Gerontol Series b: Psycholo Sci Social Sci 52B(Special):1–20. 10.1093/geronb/52B.Special_Issue.1 10.1093/geronb/52b.special_issue.1 9215354

[CR43] Tourunen A, Siltanen S, Portegijs E, Eronen J, Rantanen T, Saajanaho M (2020) Assimilative and accommodative coping and older people’s leisure activities. J Aging Health 32(7–8):778–786. 10.1177/0898264319852002 31156014 10.1177/0898264319852002

[CR44] Tourunen A, Siltanen S, Saajanaho M, Koivunen K, Kokko K, Rantanen T (2021) Psychometric properties of the 10-item Connor–Davidson resilience scale among Finnish older adults. Aging Ment Health 25(1):99–106. 10.1080/13607863.2019.1683812 31703533 10.1080/13607863.2019.1683812

[CR45] Vaishya R, Misra A, Vaish A, Ursino N, D’Ambrosi R (2024) Hand grip strength as a proposed new vital sign of health: a narrative review of evidences. J Health Popul Nutr 43(1):7. 10.1186/s41043-024-00500-y 38195493 10.1186/s41043-024-00500-y PMC10777545

[CR46] van Kessel G (2013) The ability of older people to overcome adversity: a review of the resilience concept. Geriatr Nurs 34(2):122–127. 10.1016/j.gerinurse.2012.12.011 23332474 10.1016/j.gerinurse.2012.12.011

[CR47] von Bonsdorff MB, Kokko K, Salonen M, von Bonsdorff ME, Poranen-Clark T, Alastalo H, Kajantie E, Osmond C, Eriksson JG (2019) Association of childhood adversities and home atmosphere with functioning in old age: the Helsinki birth cohort study. Age Ageing 48(1):80–86. 10.1093/ageing/afy153 30272114 10.1093/ageing/afy153 PMC6330068

[CR48] WHO. (2023, March 31). *Depressive disorder (depression)*. https://www.who.int/news-room/fact-sheets/detail/depression

[CR49] Ylihärsilä H, Kajantie E, Osmond C, Forsén T, Barker DJP, Eriksson JG (2007) Birth size, adult body composition and muscle strength in later life. Int J Obesity 31(9):1392–1399. 10.1038/sj.ijo.0803612 10.1038/sj.ijo.0803612 17356523

